# The tool in the brain: apraxia in ADL. Behavioral and neurological correlates of apraxia in daily living

**DOI:** 10.3389/fpsyg.2014.00353

**Published:** 2014-04-23

**Authors:** Marta M. N. Bieńkiewicz, Marie-Luise Brandi, Georg Goldenberg, Charmayne M. L. Hughes, Joachim Hermsdörfer

**Affiliations:** ^1^Lehrstuhl für Bewegungswissenschaft, Technische Universität MünchenMünchen, Germany; ^2^Graduate School of Systemic Neurosciences, Ludwig-Maximilians-Universität MünchenMünchen, Germany; ^3^Klinik für Neuropsychologie, Städtisches Klinikum MünchenMünchen, Germany

**Keywords:** apraxia, action disorganization syndrome (ADS), activities of daily living (ADL), tool use, cerebrovascular accident (CVA), quality of life, stroke patients

## Abstract

Humans differ from other animals in the way they can skilfully and precisely operate or invent tools to facilitate their everyday life. Tools have dominated our home, travel and work environment, becoming an integral step for our motor skills development. What happens when the part of the brain responsible for tool use is damaged in our adult life due to a cerebrovascular accident? How does daily life change when we lose the previously mastered ability to make use of the objects around us? How do patients suffering from compromised tool use cope with food preparation, personal hygiene, grooming, housework, or use of home appliances? In this literature review we present a state of the art for single and multiple tool use research, with a focus on the impact that apraxia (impaired ability to perform tool-based actions) and action disorganization syndrome (ADS; impaired ability to carry out multi-step actions) have on activities of daily living (ADL). Firstly, we summarize the behavioral studies investigating the impact of apraxia and other comorbidity syndromes, such as neglect or visual extinction, on ADL. We discuss the hallmarks of the compromised tool use in terms of the sequencing of action steps, conceptual errors committed, spatial motor control, and temporal organization of the movement. In addition, we present an up-to-date overview of the neuroimaging and lesion analyses studies that provide an insight into neural correlates of tool use in the human brain and functional changes in the neural organization following a stroke, in the context of ADL. Finally we discuss the current practice in neurorehabilitation of ADL in apraxia and ADS aiming at increasing patients’ independence.

## INTRODUCTION

Left brain damage caused by ischemic or hemorrhagic stroke is the most frequent neurological correlate of apraxia (Goldenberg, 2013). However, features of apraxic behavior can be also observed in numerous other neurodegenerative disorders (such as Parkinson’s disease, Alzheimer’s disease or posterior cortical atrophy; Bohlhalter and Osiurak, 2013) or occur as an effect of anoxia (Sirigu et al., 1995) and herpes encephalitis (Sirigu et al., 1991). Apraxic behavior in tool use is primarily attributed to the impaired or lost access to the tool related knowledge, concepts of use and problem solving (Goldenberg, 2013). Patients frequently show compromised ability to carry on everyday activities and often show action disturbances leading to safety hazards after dismissal from hospital units (Hanna-Pladdy et al., 2003). Such slips might involve attempts to use a knife in a wrong orientation to cut a slice of bread, bite a toothbrush instead of applying a brushing movement inside the mouth, toy with boiled water or tear the teabag to make a cup of tea. Problems with sequential tasks, concepts of use and smooth execution on the spatiotemporal level cannot be attributed to the deficit of function on the ipsilesional hand of patients. Patients are not able to perform the task even with the contralesional limb which might have preserved motor functionality.

The purpose of this review is to present a comprehensive summary of the research investigating apraxia syndrome following a cerebrovascular accident (CVA) and its influence on independence during activities of daily living (ADL). First, we provide a systematic overview of the behavioral research investigating impact of apraxia on three basic areas of object and action related abilities: sequencing of action, tool and gesture knowledge and spatiotemporal features of the movement, in the context of basic needs of independence. A particular focus is placed on research investigating the influence of those functions on ADL such as food preparation, personal hygiene, grooming and use of household appliances, or housework tools. The second part of this review is dedicated to the cut-edge neuroimaging research, demonstrating how multi-faceted the neural basis of tool use and ADL is as well as the current state of the art.

## DEFINITION OF APRAXIA

The most commonly used definition of apraxia was coined by Rothi and Heilman (1997) which states: “Apraxia is a neurological disorder of skilled movement that is not explained by deficits of elemental motor or sensory system.” In other words, apraxia is considered as being independent from other stroke comorbidity symptoms such as hemiplegia (loss of proprioception and motor control over limb on one side) or visual deficits such as hemianopia or neglect. However, as discussed in the penultimate section of this review, comorbidity symptoms occurring as a consequence of CVA contribute to overall ADL in a substantial manner and might even be difficult to disentangle with apraxic features. Until recently, a vast number of clinicians and researchers used the original postulation by Hugo Liepmann (a German pioneer in apraxia research) and distinguished three separate types of apraxia: ideational, ideo-kinetic (or ideomotor), and limb-kinetic (Goldenberg, 2003, 2013). Ideational apraxia refers to an inability to use familiar tools that were previously handled in an effective and purposeful manner; choosing the right object for a required action goal and carrying out multi-step naturalistic action (Goldenberg, 2013). The second category, namely ideo-kinetic apraxia, described compromised ability to pantomime actions; mimicking tool use without holding object, and/or difficulty with gesture production. In the literature, gesture production is usually divided into transitive and intransitive acts. Transitive gestures relate to object use, showing how one would use an object, whereas intransitive gestures refer to non-tool related movements, such as waving goodbye or giving someone the thumbs up. Thus, patients were reported to be unable to produce gestures that would mirror the relevant semantic representation they wished to convey (Hogrefe et al., 2012). Interestingly, even if apraxic patients attempt to operate the tool in a goal-directed fashion, they might do it in a spatiotemporally erratic manner (Poizner et al., 1995; Hermsdörfer et al., 1999; Laimgruber et al., 2005; Randerath et al., 2010). These errors are reminiscent of “limb-kinetic apraxia,” which was introduced to describe hesitation and disrupted smoothness of the movement when operating tools (both multiple and single) or disruptions of fine and precise movements, but affects only the limb opposite to the lesion (Heilman et al., 2000). To summarize, the main cognitive domains affected by apraxia comprise of the use of tools (multiple and single) and gesture production.

## DISAMBIGUATION AND COMMON GROUND BETWEEN APRAXIA AND ACTION DISORGANIZATION SYNDROME

As previously mentioned, apraxia, since the work of Hugo Liepmann, is usually linked to left brain damage (Goldenberg, 2013). Original descriptions (i.e., by Pick) of ideational apraxia were inclusive of disturbances in multi-step action performance (Goldenberg, 2013). A plethora of research demonstrates that patients suffering from right brain lesions also show disruption in terms of naturalistic action organization, referred to as action disorganization syndrome (ADS; Schwartz et al., 1999; Forde et al., 2004; Hartmann et al., 2005). ADS is a term used to describe compromised ability to sequence fixed chains of actions in an appropriate manner in relation to any naturalistic action (Humphreys and Forde, 1998). However, the differentiation between ADS and apraxia (especially ideational) is disputed. Therefore apraxia and ADS can be described under the umbrella term “apraxia and action disorganization syndrome” (AADS; Humphreys and Forde, 1998). Therefore in this review we incorporate studies investigating ADS, especially since patients with left brain damage also show difficulties with sequencing of action subtasks (Goldenberg, 2013). Probably the most puzzling element in the investigation of AADS is the lack of consistent evidence as to which brain lesions are related to the designated action problems.

## EPIDEMIOLOGY

The epidemiology of AADS was most recently reported by Bickerton et al. (2012). Approximately 46% of patients, who suffered from a first CVA were identified as symptomatic of AADS (within 6 weeks from CVA, 231 participants) based on the neuropsychological assessment (Birmingham Cognitive Screen). The criterion was impairment on one of four praxis tasks: pantomime, tool use during multi-step actions, gesture recognition or imitation. Furthermore, in the same study around 52% of those patients have shown persistent signs of AADS that did not diminish with the course of neurorehabilitation (24% of the initial sample). Previous reports, which solely focused on left hemisphere stroke survivors, estimated a rate of ideo-kinetic apraxia occurrence at approximately 30% (De Renzi, 1989). Donkervoort et al. (2001) had found that around 28% of all CVA survivors in the Dutch rehabilitation centers and 37% of nursing homes, show persistent signs of apraxia and therefore compromised ADL independence. In a later study, Donkervoort et al. (2006) stated that 88% of patients diagnosed clinically, in the acute stage with features of apraxia, were still apraxic 20 weeks post first measurement (100 days after CVA). Importantly, greater improvement over the course of rehabilitation was observed in patients that initially have had more severe deficits, whereas those with mild impairments tended to improve to a (clinically) less significant extent (measured with Barthel Index; Mahoney, 1965). Donkervoort et al. (2006) concluded that apraxia is a persistent impairment and has a negative effect on ADL. In a similar vein, Smania et al. (2006) demonstrated that apraxia is negatively correlated with the ADL independence, based on responses from patients and caregivers. On the contrary, De Renzi (1986) reported that in natural setting apraxic features are less salient due to the contextual cueing. In other words, if a patient in the hospital or lab setting has a difficulty with a simple gesture production, the same individual might still be able to perform the gesture whenever prompted by the environment (for example, to wave goodbye). Environmental information therefore has the potential to provide additional cues to promote selection of an appropriate motor program (Hermsdörfer et al., 2006). Although there is a lot of theoretical evidence supporting this view, there is no scientific ground yet to support this stance.

## USE OF ADL SCALES IN AADS

Several scales are commonly used by the clinical professionals for the assessment of ADL independence in neurological patients. Such scales are usually based on self-report or questionnaire (Barthel Index of ADL or Bristol ADL Scale; Mahoney, 1965; Bucks et al., 1996) or observation of action performed during clinical assessment (e.g., E-ADL, TULIA, NE-ADL; Gladman et al., 1993; Graessel et al., 2009; Vanbellingen et al., 2010). Those assessment tools are used not only to aid the clinical diagnosis of patients’ impairments, but also, if not primarily, to monitor efficacy of interventions to foster independence in cohort studies or clinical trials for example. Therefore the application of those scales in the clinical setting is common. Moreover, some studies have attempted to predict the speed and extent of patients’ recovery based on the overall score. For instance, Barthel Index scores measured within the approximately 3 weeks of CVA were found to be accurate predictors of compromised ADL independence in 6 months post CVA (Nakao et al., 2010). Similarly, a recent study by Bickerton et al. (2012) has noted a correlation between a multi-step action task execution and Barthel Index. Nonetheless, the assessment scales and neuropsychological batteries do not capture fully the apraxic problems patients might encounter during their daily life. Hence, relevant behavioral studies were selected for the purpose of this review to shed a light on the spectrum of difficulties that can be observed in those patients during ADL.

## BEHAVIORAL STUDIES

Most of the behavioral studies investigating apraxia following CVA focus on behavioral data with qualitative error categorization (Foundas et al., 1995; Schwartz et al., 1999). As such, the most predominant methodology includes video recordings of patients’ performance and then arbitrary classification of action errors. Setting aside the original descriptions and attempts to classify apraxia, for the purpose of this review, we can distinguish three major dimensions of action performance where apraxic features can be identified. The first one refers to sequencing problems during ADL and links to the description of ADS, compromised ability to perform subsequent actions in the correct temporal order with spatial constraints, in order to achieve an action goal (pack a lunchbox; Humphreys and Forde, 1998). For example, if one attempts to make a cup of tea, common error would involve putting cold water, not previously heated in a kettle, straight into the mug (omission error). The second area that will be discussed in this review refers to conceptual errors that might lead to the selection of the inappropriate motor plan. For example, with reference to the previously used example of tea making, one can use coffee grains instead of tea bags (substitution error; Goldenberg, 2013). In a similar fashion, communicative gestures might be misused or misunderstood. Finally, other mistakes might occur on the spatiotemporal dimension, even if the right tool is selected for action. The handling of the tool might not be adequate in terms of movement orientation, applied speed of the movement or grasp (Laimgruber et al., 2005; Randerath et al., 2010). For example, an apraxic individual might be unable to open the kettle lid during an attempt to make a cup of tea.

## SEQUENCING PROBLEMS

Daily activities rarely rely on single tool actions which require only one tool-object interaction. The majority of the actions we perform involve multistep actions leading to an action goal. The achievement of the action is comprised of the different action subgoals, constituting to chains of different activities (Goldenberg, 2013). To perform a coherent action (i.e., make a sandwich), different steps need to be organized within certain constraints of time and space (Goldenberg et al., 2001). For example, even if the individual action step is performed in a correct manner, the temporal position in the sequence chain might be out of place, in effect, leading to failure in achieving the action goal. Referring again to making a cup of tea, a person might put the kettle on, having not previously put the water inside or using another example: brush their teeth having not put the toothpaste on. Usually those errors refer to the temporal organization of the action sequence, but are not related to the personal context of actions. The overall execution of specific sequences during ADL varies interpersonally and relies on personal abilities and preferences (Land, 2006; Goldenberg, 2013; Hughes et al., 2013). Therefore, the scientific investigation of ADLs is inherently burdened with a high level of complexity of analysis and must permit a certain level of homogeneity between examined subjects. For example, healthy adults might perform an action of making a cup of tea in a variety of ways and preferences (i.e., time of the tea bag being dipped in the mug, number of sugar cubes inside) with some other sequences being constant (i.e., heating the water in a kettle before pouring it in the mug), in order to achieve an action goal (make a cup of tea). Hence certain sequences are always fixed, whereas others show a high level of inter-subject variability (Hughes et al., 2013). If the error occurs in the fixed chain of sequences, it leads to the failure to achieve the task goal and is not recoverable until the next attempt (pouring cold water into the mug with teabag inside). If however the error occurs in the “not-fixed” chain of activities, it might be recoverable.

The most frequent sequencing error in terms of action performance is the omission error, which refers to omitting a step before another one (Schwartz et al., 1999). For example, a patient might put a piece of paper into an arch file before using the hole-punch. In addition, more general sequence errors are when the patients perform something in the wrong order. Such an instance would be putting or adding an extra sequence or ingredient (addition) that is not needed or that is repeated (perseveration error; Rumiati et al., 2001). In another scenario, a subtask might be performed too early in the chain of sequences (anticipation error). An example of sequence addition error would be folding a piece of paper before putting it into the arch file in a document filing task. Another type of addition, based on the use of additional objects or ingredients (in food related tasks) would be (using the previously mentioned example) putting a piece of scotch tape on the top of the paper. In sum, CVA subjects might engage in sparse subtasks that are not relevant in the context of achieving the action goal. In the same task, a perseveration error would describe repetition of the previously accomplished subtask, such as making more punch holes than necessary. There is a plethora of research that has attempted to capture the most common error occurrences in naturalistic action performance with different types of error patterns. However, the results show some incongruence between the terminology used and the classification of errors (see Goldenberg, 2013, Chap. 9, for review on this issue). Previously mentioned omission errors reach an approximate ratio of 40–50% for all action errors (Schwartz et al., 1999; Bickerton et al., 2007). Importantly, the tendency to skip a step that is necessary for achieving the action goal seems to be linked to the level of familiarity with the object. Novel object, which are not familiar to patients seem to elicit the highest number of those errors (Bickerton et al., 2007). Other authors also point out the prevalence of these types of action errors, but they use different terminology to describe it, namely sequence error (De Renzi and Lucchelli, 1988) or action anticipation (Rumiati et al., 2001). **Table 1** presents an overview of research describing the sequencing errors related to the ADL in stroke survivors studies.

**Table 1:** Summary of studies on sequencing errors related to the ADL in AADS.

Source	Participants	Task	Main results
Bickerton et al. (2007)	ADS patient (*N* = 1); patients with brain lesions (*N* = 4); age- and sex matched controls (*N* = 5)	Making a cup of tea/coffee/toast/sandwich, wrapping a gift, write and post a letter, packing a lunchbox, putting an article from a magazine into a file	ADS patient made more omission steps with unfamiliar than familiar objects compared to controls (2 and 0.5 errors, respectively)
Bickerton et al. (2012)	RBD and LBD (*N* = 635), age- and sex matched controls (*N* = 100)	Mounting a torch and switching on light (MOT task)	No differences between LBD and RBD in MOT score, low but consistent correlation between MOT and Barthel Index (*r* = 0.29) and Nottingham Extended ADL scale (*r* = 0.32)
Buxbaum (1998)	Patients with LBD (*N* = 16)	Wrapping a gift, making toast, packing a lunchbox	Ratio of errors: omissions (44%), sequence errors (27%)
Humphreys and Forde (1998)	ADS patient (*N* = 2)	Wrapping a gift, posting a letter, making toast/sandwich/cup of coffee, preparing cereal, tooth brushing, shaving, painting wood	Omissions (24%), sequence errors (40%); patients better with shorter than with longer tasks
Schwartz et al. (1999)	Patients with RBD (*N* = 30)	Wrapping a gift, making toast, packing a lunchbox	Omissions (47%), sequence errors (19%)
Sunderland et al. (2006)	Patients with right and left hemisphere stroke (*N* = 8), five RBD, four LBD	Dressing	76% LBD demonstrated a planning problem (dressing first the non-paretic arm), RBD attentional and spatial problems (e.g., finding sleeve opening), 16% of RBD did not push sleeve over the paretic elbow

As reported in **Table 1**, there is a substantial body of research attempting to capture problems with sequencing of ADL in CVA patients. Different classifications are proposed by many research groups, but not all of them fit to every ADL, due to the variation in the fixed or not fixed action chains. However, most authors agree that problems with the organization of particular subtasks should be referred to as sequence errors, with subclasses, such as addition errors or anticipation, or without (De Renzi and Lucchelli, 1988; Schwartz et al., 1999; Rumiati et al., 2001; Goldenberg, 2013). In the seminal study by Foundas et al. (1995) conducted on 10 patients with unilateral left hemisphere CVA no error classification was used. Authors observed the lunchtime behavior (via video taping) on the hospital ward and divided the overall meal organization into three phases: preparatory, eating and clean up. Only 20% of CVA patients proceeded with all three phases of the meal and only 40% demonstrated preparatory behavior. In comparison to all healthy age-matched controls engaged in preparation of the meal, and 80% in the clean-up phase. In addition, patients used fewer tools (cutlery) than controls and shown different pattern of food consumption (consuming one ingredient in a sequential fashion or drink a glass of refreshment at once) in comparison to controls (who preferred to mix different ingredients and take small sips of drink).

## CONCEPTS OF USE AND GESTURE KNOWLEDGE

On the cognitive level, the knowledge about concepts of use can be referred to as both functional knowledge (Sirigu et al., 1995) and the ability for mechanical problem solving (Goldenberg and Hagmann, 1998; Osiurak et al., 2009). Functional knowledge specifies the typical purpose, recipients, and manner of using distinct types of tools (Sirigu et al., 1991; Hodges et al., 2000; Rumiati et al., 2001). This type of expertise embraces global motor concepts, inclusive of the recipient of the action, relevant manipulation, and tool selection for the desired action goal (Goldenberg, 2013). For example, a hammer can be used to put a nail into a block of wood through forceful strokes. The knowledge necessary to achieve this goal includes: choosing the right tool from the toolbox (hammer); knowing how to position the nail in the block of wood and knowing what movement to apply. There is, however, controversy whether the kinematics of actions and the formation of adequate hand postures are stored in a separate compartment of semantic memory as “manipulation knowledge” or are derived from structural properties of tools by mechanical problem solving (Goldenberg and Spatt, 2009; Osiurak et al., 2009; Kalénine et al., 2010). Patients with loss of functional tool use knowledge may be able to infer the function of the object from their structure (Goldenberg, 2009). In the modern type of devices however, such as technically advanced coffee machines with capsules, patients are not able to deduce (use mechanical problem solving) how to operate the device based on its physical structure. Therefore those types of the devices (such as tablets or smart TV) might be almost impossible to operate for apraxic individuals (Hartmann et al., 2005).

In principle, ADL can be divided into multiple tool use and single tool use actions (Goldenberg, 1996, 2013). For example, making a cup of tea would be an example of complex and multiple tool based action. On the contrary, fixing a loose screw would be based on single tool use, namely a screwdriver. One of the common errors noted in the literature is mislocation or misplacing of the tool (De Renzi and Lucchelli, 1988; Schwartz et al., 1999) or spatial error as described by Humphreys and Forde (1998). De Renzi and Lucchelli (1988) tested 20 patients in the tool use and pantomime paradigm. Among other errors, author’s differentiated mislocation as appropriate action carried out in the spatially inadequate place. For instance, patients were able to strike a match, but tried to lit the wrong side of the candle. Misuse of the tool has also been identified by De Renzi and Lucchelli (1988) and Rumiati et al. (2001). Misuse can be defined as use of object in conceptually inappropriate way, i.e., rubbing candle onto the table, or handling object by the wrong end (De Renzi and Lucchelli, 1988). All of the error classifications mentioned refer to the impaired ability to handle the tool in a relevant manner (i.e., also include uncomfortable grasp of the tool). Other research also reports wrong object selection (Humphreys and Forde, 1998; Goldenberg, 2009) or object substitution (Schwartz et al., 1999). Humphreys and Forde (1998) tested two patients with features of AADS on ten ADL tasks (see **Table 1**). In the tea making task, one of the patient demonstrated repetitive errors of pouring milk into the teapot rather the mug. Authors referred to it as semantic error, specific for object selection. Schwartz et al. (1999) tested 30 patients with right hemisphere lesions following CVA on three ADL tasks (making a toast, wrapping present, and packing lunchbox). Object substitution was defined as correct movement performed with wrong object, i.e., putting a slice of bread on a hot plate instead inside the toaster. In addition, misestimation errors, i.e., too little or too much of one ingredient, were introduced in studies looking into food related behavior (Foundas et al., 1995). For example, patients were reported to put too little food on their plate and fork during daily lunchtime behavior or making a toast (Foundas et al., 1995; Schwartz et al., 1999). Importantly, the differences within classification of the errors are arbitrary and do not have a consequence on the overall understanding of the difficulties patients exhibit with ADL. Patients might choose the wrong tool for an action, for example, picking up a screwdriver to connect two sheets of paper together. In many occasions the difficulties with access to the adequate motor concepts do not manifest themselves directly but are observable as perplexity or toying behavior. Those errors are not explicitly categorized separately by all researchers (e.g., Schwartz et al., 1999). Perplexity refers to pauses in movement, or inefficient manipulation. For example, the patient might pick up objects and then set them back on the work surface and cease further trials to accomplish the task goal. Toying, on the other hand describes handling an object in a non-purposeful fashion. One measure that can capture those behaviors, aside from video scoring of conceptual errors committed by patient, is movement time for the task completion.

## SPATIOTEMPORAL FEATURES OF APRAXIA

A seminal study by Foundas et al. (1995) on meal preparation, has revealed that left brain damaged patients were less successful in the overall organization of the preparation of meals and that the “correct tool actions” measure significantly correlated with the apraxia score (Florida Apraxia Battery, Rothi et al., 1992). The overall time difference between patients (slightly prolonged) and healthy controls was however not statistically significant. Spatiotemporal errors of movement execution have been documented mostly during pantomime of tool us but have also been found during real tool use (Hermsdörfer et al., 2006). Spatiotemporal errors in the task performance can have a discrete demonstration when the individual is performing an action in a kinematically incongruent manner, which might or might not be observable with the naked eye even for a non-expert viewer. Poizner et al. (1995) and Clark et al. (1994) have demonstrated that apraxic patients with left brain damage suffer from impaired joint coordination and imprecise plane of motion, along with trajectory shape in a bread slicing task. In addition, impaired coupling between the hand speed and trajectory shape was identified. However, it remains open whether these kinematic irregularities reflect deficits of motor-coordination directly or are due to slow and hesitating movement execution due to conceptual problems with planning the action (Hermsdörfer et al., 2006). In other words, impaired movement on the spatiotemporal dimension might be a reflection of compromised movement planning, but not be a feature of limb apraxia. In a seminal study by Laimgruber et al. (2005) left brain damaged patients were found to demonstrate a prolonged adjustment phase before grasping a glass of water, whereas right brain damaged patients showed a decreased velocity of the movement. Speed deficits were also found in the sawing tasks in left brain patients in comparison to age-matched controls (Hermsdörfer et al., 2006). Other variables such as prolonged reaction times and reduced amplitude of the movement were reported for the hammering and scooping movement actions in left brain damaged patients (Hermsdörfer et al., 2006, 2012). Deficits of spatiotemporal aspects of movement execution may be directly or indirectly related to apraxia as indicated above, but also may be indirectly related to spatial deficits such as neglect or they may also be independent consequences of damage to the motor-dominant hemisphere (Hermsdörfer et al., 2012). Randerath et al. (2010) has found that left brain damage patients show impairment in the grasping movements during single tool use. In comparison to healthy age-matched controls, patients demonstrated significantly higher percentage of non-functional grasps of the tools’ handles. The impaired grasp was predominately followed by erratic demonstration of the tool use. In the real life scenario, those spatiotemporal deficits might result in mishandling of the object, leading to safety hazards, or frustration (Hanna-Pladdy et al., 2003). In the next section we will present an overview of the neural underpinnings of ADL and apraxia, which will shed more light on the complex organization of human tool use.

## THE NEURAL BASIS OF ADL

This section of the review is organized in a similar fashion to the behavioral part, with division of the studies to the sequencing of subgoals of ADL, then conceptual understanding and finally spatiotemporal features of ADL. To provide an insight into the neural correlates of ADL and apraxia, we present neuroimaging studies with healthy subjects followed by lesion analyses with apraxic patients.

## HEALTHY ADULT STUDIES

We aim to discuss the neural basis of ADL by including functional neuroimaging studies on viewing, understanding, imagining, pantomiming and executing ADL and single tool use in healthy adults. Furthermore only studies on sequencing actions, tool knowledge and the spatiotemporal features of actions with tools are summarized and visualized here. For the visualization of the neural correlates of these three aspects of ADL, we used the GingerALE toolbox (Eickhoff et al., 2009, 2012) for conducting a meta-analysis. The relevant peak coordinates (in Talairach space) from whole brain analysis were entered separately for the three aspects of ADL. The main aim of this analysis was to provide a descriptive visualization of the activation patterns found in the relevant studies. Therefore a relatively low threshold (*p* < 0.05 FDR corrected) was used to create the ALE images (Laird et al., 2005). The toolbox Mango (Designed and developed by Jack L. Lancaster and Michael J. Martinez) was used to map these thresholded ALE images of all three categories on a rendered brain and to locate the visualized brain areas.

## ACTION SEQUENCING

**FIGURE 1:**
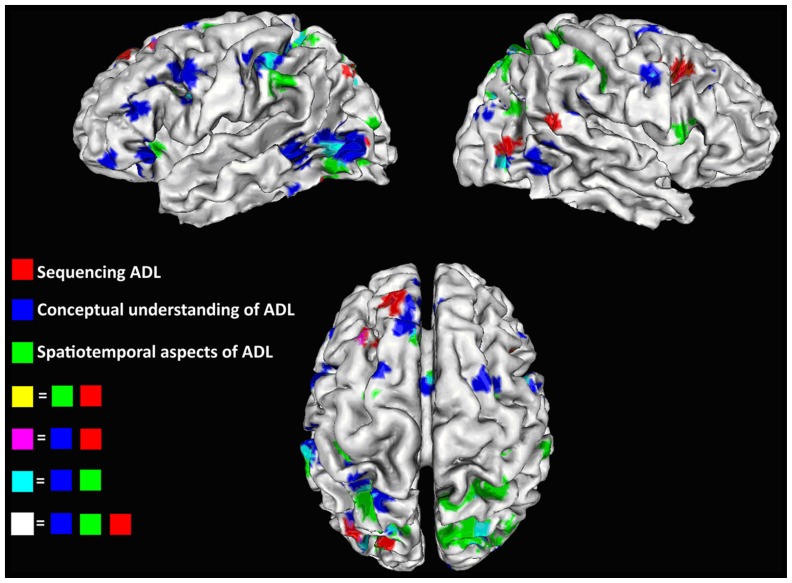
**ALE images for studies focusing on action sequencing in red, conceptual understanding of ADL in blue, and spatial orientation of ADL in green; Overlays are depicted in purple (blue + red), light blue (blue + green), and white (all three)**. Images are produced with the GingerALE toolbox (Eickhoff et al., 2009) and have a threshold of *p* < 0.05 with FDR correction.

As described previously, patients suffering from AADS show difficulties with sequencing multi-step actions and single tool use. The neural underpinnings of action sequencing in ADL are not yet fully understood. Only a few studies have so far investigated brain regions relevant for sequencing sub-actions of ADL. The most seminal studies in the area were conducted by Schubotz et al. (2012) and Zacks et al. (2001). In these studies subjects had to watch videos depicting different ADL with multiple sequences (for example washing the dishes or ironing a shirt) and had to detect the time borders when each of the sub-actions had commenced. In addition, Weiss et al. (2006) has analyzed the processing of errors in the sequential structure of ADL. Here, subjects had to watch videos of ADL including, for example, pouring a glass of water and drinking it, lighting a candle with matches or affixing a stamp on a letter. These videos were either correct or included errors in the order of sub-actions, which the subjects had to detect. In summary the brain areas relevant for processing the separation and ordering of sequences in ADL cover areas of the frontal, parietal, temporal and occipital cortex. More precisely, these areas were pinpointed to the inferior and middle frontal gyrus, angular gyrus and adjacent precuneus, middle temporal gyrus, fusiform gyrus, and middle occipital gyrus of the left hemisphere. Additional clusters can be seen in the right middle frontal gyrus, middle occipital gyrus, precuneus, inferior and superior temporal gyrus, and fusiform gyrus. The ALE image depicting results from those studies is shown in the **Figure 1** in red.

## CONCEPTUAL KNOWLEDGE OF TOOL USE

To get an overview of the neural basis of the conceptual knowledge in the context of ADL and single tool use, we summarized studies investigating how the knowledge of tools and their function is coded in the brain. We included studies comparing correct versus incorrect use of a tool dependent on the context (Mizelle and Wheaton, 2010; Wurm et al., 2012) and studies comparing tool actions of familiar compared to unfamiliar tools (Menz et al., 2010). Exemplary stimuli used in these studies were videos showing actions like punching holes in paper (Wurm et al., 2012) or images and animations of using a hammer (Menz et al., 2010; Mizelle and Wheaton, 2010). In addition, two other studies were included (Manthey et al., 2003; Hoeren et al., 2013), which evaluated both the conceptual understanding of ADL and also the processing of the spatial organization of actions separately. The latter aspect will be discussed in the next paragraph. In the study of Manthey et al. (2003) subjects had to watch videos with ADL and detect object related errors (for example: pour coffee in a glass instead of a cup), or movement errors in the viewed actions (for example: open a bicycle lock but holding the key transverse to the lock). In the Hoeren et al.’s (2013)study subjects were asked to decide, if the object used in an action fits to the context (for example: a cake lifter is used for cake not for a steak in a pan), or if the hand position is correct to perform the known action with the object. In all studies subjects had to show a conceptual understanding of ADL to perform the different tasks. More specifically, the participants had to know the purpose of the actions they saw and the function of the tool used in the actions. Findings from these studies have demonstrated that understanding and tool use function in ADL recruits a wide (mostly left lateralized) network covering frontal, parietal, temporal and occipital centers. Main activation sites were reported on the left hemisphere in the frontal cortex and include inferior, middle and superior frontal gyrus; in the parietal cortex clusters covering anterior to posterior part of the intraparietal area, angular and supramarginal gyrus, and superior parietal lobule activations were reported. Activations in the middle and superior occipital gyrus were found in the occipital cortex. In the temporal lobule, activation patters mainly covered the posterior part of the middle and inferior temporal gyrus and the fusiform gyrus. In the right hemisphere, activation was pinpointed to the middle, superior and inferior frontal gyrus in the superior parietal lobule and anterior part of the intraparietal area, as well as in middle temporal, inferior occipital, and fusiform gyrus. The activation in the right hemisphere is partly homologous to the left areas, but the overall activation pattern comprises less brain areas. A summary of brain network recruitment reported in the mentioned studies is shown in **Figure 1** in blue.

## SPATIOTEMPORAL ORGANIZATION OF MOVEMENTS

As mentioned in the previous sections of this review, the third component of ADL (following the sequencing of the actions and conceptual knowledge) concerns the tool manipulation necessary to achieve the intended goal and incorporates spatiotemporal features of the movement. This includes grasping the tool in a correct way and moving it accordingly across space. Functional imaging studies have analyzed the brain areas relevant for selecting the correct grip for tool usage during ADL (Valyear and Culham, 2010; Vingerhoets et al., 2010; Hoeren et al., 2013) or the spatial organization of the movement (Manthey et al., 2003; Weiss et al., 2006; Yoon et al., 2012). The neural correlates of this component are more bilateral and mainly include parietal, frontal and occipital areas of both hemispheres. These include the superior and inferior parietal regions, the area close to the posterior part of the intraparietal area and the parieto-occipital sulcus (parieto-occipital junction), premotor cortex and the middle occipital gyrus in both hemispheres. In addition, studies mentioned above have found that the ventral premotor area is relevant in the right hemisphere and the anterior insula in the left. In general, it can be mentioned that most clusters relevant for grip selection and the spatial monitoring of tool use mainly cover regions related to the dorso-dorsal pathway as described by Binkofski and Buxbaum (2012).

## SUMMARY OF THE FUNCTIONAL IMAGINING HEALTHY ADULTS SECTION

Investigation of main cortical activation sites of all three aspects of ADL yields the involvement of a wide neural network including frontal, parietal and temporal centers (**Figure 1**). Overlaps were found between the different maps for regions processing conceptual and spatial information of tool use and ADL including frontal clusters in the dorsal and ventral premotor areas, in the anterior cingulate cortex, in the parietal lobe along the intraparietal area, the superior parietal lobule, the supramarginal gyrus, around the parieto-occipital sulcus and in the inferior temporal gyrus of the temporal lobe of the left hemisphere. We have found less overlaps in the right hemisphere, which comprise parts of the parietal lobule, precentral gyrus and inferior temporal gyrus. In addition, we report a partial congruency between clusters from sequencing studies and studies focusing on knowledge of tool use. These are associated with activation in the dorsal premotor area, posterior part of the intraparietal area, middle occipital gyrus and fusiform gyrus of the left hemisphere. In summary, ADL and single tool use are complex tasks with multiple aspects to be processed which recruit wide brain networks. Importantly it has to be stated that the neural bases of the three aspects discussed here cannot be clearly separated in actual tool use but need to be integrated to perform ADL. Evidence supporting the importance of the mentioned network is also provided by studies focusing on the neuronal basis of actual tool manipulation, which covered more general or other aspects of tool use (Hermsdörfer et al., 2007; Imazu et al., 2007; Gallivan et al., 2013). In addition, studies on pantomime of tool use also support the present findings (Moll et al., 2000; Inoue et al., 2001; Johnson-Frey et al., 2005; Króliczak and Frey, 2009; Vingerhoets et al., 2011).

## LESION ANALYSIS IN PATIENT WITH BRAIN DAMAGE

Another method that sheds light on the neuroanatomical correlates of tool use is a lesion symptom analysis in CVA patients. In those studies, behavioral measures are correlated with lesion sites to create statistical brain maps showing the location of lesions closely linked to a behavioral deficit. Compared to the studies with healthy subjects, studies including lesion analysis focusing on executing or recognizing ADL are relatively rare (Pazzaglia et al., 2008; Goldenberg and Spatt, 2009; Randerath et al., 2010; Hermsdörfer et al., 2013; Kalénine et al., 2013). Therefore, a differentiation of action sequencing, conceptual understanding and spatiotemporal aspects of tool use, to the same extent as in healthy subjects or purely behavioral clinical studies, is limited. Hence, we aim to concentrate on studies including tasks testing performance of actual tool use and understanding or recognition of goal directed actions (Pazzaglia et al., 2008; Goldenberg and Spatt, 2009; Hermsdörfer et al., 2013; Kalénine et al., 2013). Additional information is given on the neuronal correlates of tool grasping next to tool usage (Randerath et al., 2010) and to increase the scope on the neural basis of sequencing ADL in patients, a study focussing on the sequencing of pantomime tool use (Weiss et al., 2008) will also be mentioned here.

In a study by Goldenberg and Spatt (2009), 38 patients with left sided brain lesions, were tested to assess possible deficits in functional knowledge of tools and objects, mechanical problem solving (which was tested with the use of novel tools), and additionally the selection and usage of common tools. Impairments in these tasks were related to two major lesions sites, one around the middle frontal gyrus reaching to the inferior frontal gyrus, which was related to deficits in all three tasks, and a second lesion site in the parietal cortex, reaching from the supramarginal gyrus through the inferior parietal lobule to the superior parietal cortex. The second lesion site mainly impaired the selection and use of common and novel tools. After looking at a subset of patients with deficits in the functional knowledge of tools (but not in mechanical problem solving) Goldenberg and Spatt (2009) found an association of this selective impairment to lesions in the middle temporal gyrus.

The relation of performance in tool use and lesions of patients with left sided brain damage was also analyzed by Hermsdörfer et al. (2013). Next to pantomime and imitation tasks, the correct performance of real tool use was measured and put in relation to the patients’ lesions. In this study, low performance was also associated with parts of the inferior frontal gyrus including pars opercularis, triangularis, and insula.

As well as these two studies, which analyzed actual tool use, there are other studies focussing more on the understanding or recognition of actions. Kalénine et al. (2013) distinguish two parts of goal directed actions: action means and action outcome. The first component – dealing with “what” has to be done to achieve a goal (spatiotemporal features of the tool use) and the latter one – representing the actual outcome of the action (conceptual knowledge). Patients with left sided brain lesions, were asked to evaluate if two actions they saw in a video, were the same or different. These videos differed either in their action means or outcome. The performance of this detection task was combined with information from the patients’ lesions, demonstrating a specific relation between lesions in the inferior parietal lobe with action means but not outcome. This underlines previously mentioned findings, stating the relevance of the inferior parietal lobe in processing the knowledge of what has to be done with a certain object or tool to achieve a goal.

The recognition of action related sounds and the execution of these actions was analyzed in a study from Pazzaglia et al. (2008) linking to the conceptual knowledge of tool use. Sounds of buccofacial-related or limb-related actions known from daily life had to be recognized by the patients and also executed. The lesion analysis revealed that impairment of action recognition and execution of buccofacial-related sound was mainly correlated with lesions in the inferior frontal gyrus and insula. Impaired limb-related action recognition and execution on the other side was associated with lesions in the inferior parietal lobe, supramarginal gyrus, angular gyrus, and also the inferior frontal gyrus. A stronger involvement of tool related parietal regions in limb-related action recognition, compared to buccofacial-related actions can be due to the fact that limb-related action sounds and executed actions included more tool actions, than the other condition.

Another lesion analysis including the analysis of actual tool use in patients with left sided brain damage was performed by Randerath et al. (2010). Patients had to grasp a tool and demonstrate its use for various tools with handles oriented toward or away from their body. In this study, the type of grasp (functional or non-functional) and the correct demonstration of tool use were evaluated and correlated with patients’ lesions. The main findings related an impaired grip of tools to the lesions in the parieto-occipital junction, the angular gyrus, and especially in the inferior frontal gyrus, in particular the pars orbitalis, opercularis and triangularis. An incorrect demonstration of tool use on the other side was most closely linked to lesions in the supramarginal gyrus of the inferior parietal cortex and the gyrus postcentralis. An overlap between impaired grip and incorrect demonstration of tool use was found mainly in the inferior parietal cortex. As discussed by the authors, these findings are in line with the assumptions that the function specific manipulation of tools is mainly processed in the ventro-dorsal part of the dorsal stream (Rizzolatti and Matelli, 2003; for review see Binkofski and Buxbaum, 2012). According to this theory, reaching and grasping movements are related to dorso-dorsal regions like the superior parietal lobe, caudal parts of the intraparietal sulcus, parietal-occipital sulcus and the adjacent parietal-occipital junction (Karnath and Perenin, 2005; Prado et al., 2005). The findings of Randerath et al. (2010) underline the relevance of the parietal-occipital junction for correct grasping, especially for using tools.

To our knowledge, so far, only one study has performed a lesion analysis including sequencing of actions of daily living. In a study of Weiss et al. (2008) patients had to detect sequential and spatial errors in object related actions with or without the object. The main focus of the lesion analysis in this study was sequential error detection in actions without an object (pantomime of action). This analysis revealed that patients with severe problems in recognizing the correct timing sequence of an action had a common lesion site in the left angular gyrus of the parietal lobe.

**FIGURE 2:**
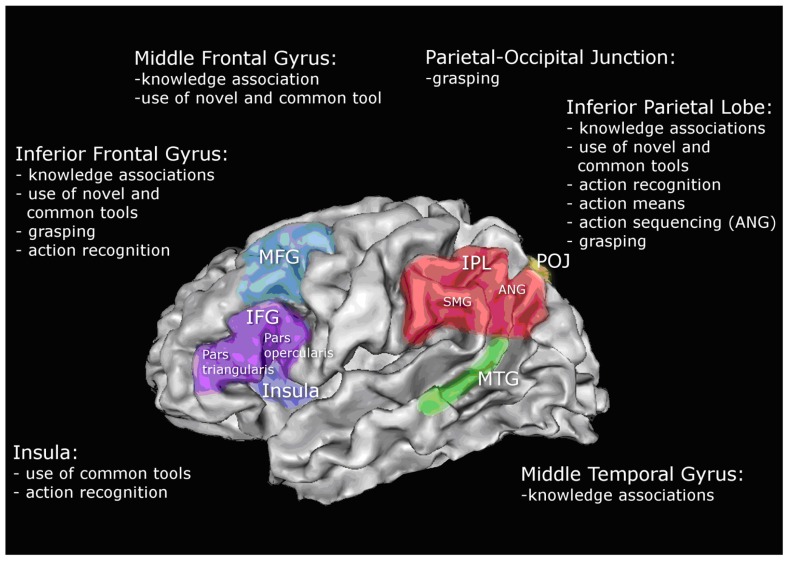
**Schematic illustration of left hemisphere associations with performance in tool use and ADL based on the reviewed studies; middle frontal gyrus (MFG); inferior frontal gyrus (IFG); inferior parietal lobe (IPL); supramarginal gyrus (SMG); angular gyrus (ANG); parietal-occipital junction (POJ); middle temporal gyrus (MTG)**.

In summary, the impairment in the recognition or performance of ADL including tool use was reported by many studies to be related to frontal lesions, especially the inferior frontal gyrus, inferior parietal lesions including supramarginal and angular gyrus and the neighboring parieto-occipital junction and lesions in the middle temporal gyrus. An overview of the affected regions and the associated tasks which were impaired, after lesions in these areas, is shown in **Figure 2**. Further evidence of the relevance of these brain regions in apraxia can be derived from lesion analyses focusing on pantomime of tool use. Again the ability to recognize pantomime of daily actions (Kalénine et al., 2010) or the execution of it (Buxbaum and Saffran, 2002; Buxbaum et al., 2005; Goldenberg et al., 2007; Hermsdörfer et al., 2013; Manuel et al., 2013) is strongly related to the already described lesion sites.

Considering the functional imaging studies on tool use and actions of daily living of healthy adults, we see a substantial overlap with the results of the lesion studies. For action sequencing, both imaging studies and lesion analysis show that the left angular gyrus plays a critical role. The conceptual understanding of tool use in ADL, on the other hand, comprises a larger network with core centers in the inferior frontal gyrus, the inferior parietal lobe and middle temporal gyrus. The neuronal processes of the spatiotemporal organization of actions in both healthy adults and also in patients were related to the posterior part of the parietal lobe including the angular gyrus, the parieto-occipital junction and the inferior frontal gyrus.

## COMORBIDITY SYMPTOMS

As mentioned before, AADS syndrome might be enhanced by other comorbidity syndromes following a stroke (Goldenberg, 2013). The research that attempts to link different types of errors to other deficits that are co-morbid to apraxia in the CVA patients is partially unfruitful. One of the problems is that it is difficult to untwine which of the symptoms contribute the most to the difficulty with task execution. Around 30% of ischemic stroke survivors suffer from cognitive impairments apart from the motor disability, affecting speech ability, vision, memory and attention (Katz et al., 1999). For example, Walker et al. (2012) has demonstrated that dressing problems in the right brain damaged patients are mostly attributed to visuospatial deficits. In a similar vein, other studies have reported that visuospatial neglect (impairment of spatial attention) is a stable predictor for the functional outcome of the rehabilitation in the post hospitalization period (Denes et al., 1982; Edmans and Lincoln, 1991; Katz et al., 1999; Jehkonen et al., 2000). Other symptoms, such as hemiparesis, amnesia, visual construction problems and language deficits were reported to lack predictive power (Jehkonen et al., 2000). Importantly, this was contested by research conducted by Wade and Hewer (1987) pinpointing hemianopia (loss of side of visual field) as a second factor for functional recovery in post-acute phase of stroke. More recent work by Paolucci et al. (1998) has stated that absence of neglect is the most important prerequisite for the promising prognosis for ADL independence. In addition, Pedersen et al. (1997) identified within the group of neglect patients that anosognosia (compromised self-awareness of own mental and physical state) is in fact a more powerful predictor of recovery in those patients. Therefore, many of the therapeutic approaches are targeted at broadening the visual field in patients suffering from hemianopia or hemineglect, through multisensory stimulations (Làdavas, 2008) or spatiomotor cueing (Kalra et al., 1997). The underlying assumption is that an effective rehabilitation plan needs to incorporate multicomponent factors and, in order to regain independence during ADL, a multifaceted approach is recommended – targeted at different neuropsychological symptoms (Katz et al., 1999). However, until now, there is no conclusive scientific evidence linking the severity of AADS with other neuropsychological symptoms, in particular, neglect. It is however clear that each of these symptoms has its own neural representation and a lesion will affect an aspect of ADL. These considerations reflect the difficulty to define a circumscribed neural network related to ADL. Rather, the network will be widespread with soft boundaries between areas directly and indirectly involved in action planning and tool use.

## CONSEQUENCES OF APRAXIA AND AADS ON ADL, RECOVERY RATE, REHABILITATION

Although the incidence of apraxia is relatively high, the common view was that apraxia recovers spontaneously (Basso et al., 1987). However, this outlook is contested by the previous work of Hanna-Pladdy et al. (2003) and Smania et al. (2006) reporting that CVA patients struggle with ADL, due to residual traits of apraxia. Therefore, rehabilitation of apraxia maintains a significant challenge for the clinicians and occupational therapy workers. The research in this matter is inconsistent and limited in comparison to the number of studies investigating behavioral and neural correlates of apraxia (Goldenberg, 2013). According to Buxbaum et al. (2008) the common treatment approach is focused on teaching compensatory techniques for ADL tasks, which allow fostering independence despite the presence of apraxia. This strategy training comprises of the errorless training and high number of repetitions for particular task or verbalisation techniques (Goldenberg, 2013). In errorless approach the therapist guides the patient through the correct sequence of ADL and prevents the occurrence of action errors. In a similar vein, Buxbaum et al. (2008) reported that committing errors during training is disruptive for the outcome of retraining, thus compensatory strategies should be based on errorless approach. Goldenberg et al. (2001) states that intensive training improves specific task performance but cannot be generalized to other activities. In other words, training has to be task specific and does not transfer to other non-trained tasks (Goldenberg and Hagmann, 1998). Interestingly, in this report the majority of patients showed a deterioration of independence during ADL when therapy was withheld (2–5 weeks training period, daily 20–40 min). Exploration training, pointing out critical features of objects, without guidance how to use them did not bring improvement in patients (Goldenberg et al., 2001). Donkervoort et al. (2001) argues that strategy training may bring a short term benefit for patients and improve the global ADL functioning, but is the most effective in conjunction with standard occupation therapy. In their study intervention was based on verbalisation techniques comprised of providing narrative to guide through the task performance. Furthermore, another approach with evidenced efficacy is based on gesture training, which is more related to pantomime function (Buxbaum et al., 2008). This training is dedicated to practicing gestures associated with tool use. Smania et al. (2000) reported significant reduction in praxis errors and gesture comprehension after 35 training sessions (50 min each). In a subsequent study Smania et al. (2006) showed retention of gains 2 months post treatment after 30 training sessions of the same length as in previous report. In both studies, limited generalization to other tasks was found, but no impact on the overall ADL independence was noted. In addition, the home environment for training was pointed out to be important factor of recovery in 8 week intervention study (Geusgens et al., 2007). Tasks should be important for daily routine and meaningful for the patient. As summarized by Goldenberg (2013) AADS is not a homogenous disorder thus therapy approaches are usually adjusted to the core manifestations. Another aspect is that even if efficacy of training is maintained, it addresses primarily the ability to use compensatory strategies promoting independence during ADL, but does not affect the “concepts of use” (Goldenberg, 2013). Furthermore, the generalisability of training one task to global impact on ADL independence is often not assessed or not found, along with limited evidence for follow-up effectiveness (Maher et al., 1991; Pilgrim and Humphreys, 1994; Ochipa et al., 1995; Goldenberg et al., 2001). Consequently there is lack of clear guidelines what period of time is the optimal for treatment of AADS, which intensity of training is recommended and how to prolong the effects of therapy. Study by Goldenberg and Hagmann (1998) suggests that effects of the intervention can be only sustained if patient continues at home training of ADL independence. Training over the period of a few weeks is feasible if outpatient clinics or day clinics are in place. This, however, is increasingly challenged in the current economic climate, due to restricted funding for the post hospitalization phase. Therefore technology driven solutions might be soon developed to provide continuity for ADL training. In addition, if restoration of the function is impossible, rapidly developing technologies might soon provide a real time “crutch” for independence for stroke survivors. Use of the assistive devices in the home environment could provide additional contextual information for the patient in the ecologically valid setting. Contextual cueing was demonstrated by Maher et al. (1991) to improve performance of a chronic patient with ideo-kinetic apraxia (case study), within 2 weeks of therapy, based on the shaping (slow withdrawal of cues) paradigm. A similar idea was posited by Buxbaum et al. (2008) discussing the possibility of using robot-assisted devices.

Current projects, which aim to provide autonomous systems of guidance for patients struggling with ADL, are primarily tailored for subjects with dementia and use the concepts of domotics (intelligent home environment). One of the projects currently under development is the COACH system, which is designed to provide assistance in hand washing action to residents of nursing home for people with dementia (Mihailidis et al., 2008). Based on computer vision the system is capable of recognizing problems with task performance. The interface provides prompts based on verbal and visual information, with the prompts adjusted to the needs of patients (for example video or auditory cues). Another project, based on similar type of modeling and solutions is TEBRA, dedicated to aid tooth brushing performance in people with dementia in the home setting (Peters et al., 2013). Finally CogWatch (www.cogwatch.eu) is a system that is currently under development, which is tailored to the needs of AADS patients. The aim is to create fully automatised computer–human interface that provides cues or prompts errors during the performance of ADL (i.e., tea making and tooth brushing). Creating an autonomous system that could aid rehabilitation of ADL in AADS group is a technological and theoretical challenge, which surely will be pursued in the further research developments and projects.

## CONCLUSION

The review summarized the most significant research conducted on the impact of AADS on the ADL in stroke survivors. Behavioral, neuroimaging and lesion studies were presented to give an overview of the current state-of-art. Taken together, CVA resulting in lesions in the left or/and right hemisphere has profound consequences on the daily independence of patients during everyday tasks such as food and drink preparation, grooming, personal hygiene, and use of everyday objects. A new approach was adopted to provide a comprehensive description of the unique features of apraxic and action disorganization disorder. The difficulties with execution of ADL were categorized arbitrarily into three components: problems with sequencing of the multi-step actions, conceptual knowledge about tool use and spatiotemporal aspects of the movement. This classification is novel in comparison to the original descriptions of AADS. However, the aim of this approach was to provide a comprehensive insight into the global picture of difficulties CVA patients might experience. Although these themes were presented separately, the evidence suggests those deficits are often intertwined on the behavioral level and also share the neural substrates. In the neural correlates section of this review, the critical role of the left angular gyrus was pinpointed in the sequencing of the multi-step actions. The neural underpinnings of conceptual knowledge were located in the inferior frontal gyrus, the inferior parietal lobe, and middle temporal gyrus. The spatiotemporal features of the execution of the ADL have been linked to the integrity of posterior part of the parietal lobe including the angular gyrus, the parieto-occipital junction and the inferior frontal gyrus. In addition, other areas that were also identified as linked to the ADL performance were discussed, with a conclusion that a wide neural network is involved in cognitive and motor aspects of action planning and execution. In the final section of this review, a strategy training approach was identified as the most efficient and common therapeutic strategy currently used in the rehabilitation of AADS.

## Conflict of Interest Statement

The authors declare that the research was conducted in the absence of any commercial or financial relationships that could be construed as a potential conflict of interest.
